# Strategies and designs for combination immune therapy

**DOI:** 10.1186/1479-5876-13-S1-K7

**Published:** 2015-01-15

**Authors:** Samir Khleif

**Affiliations:** 1 Georgia Regents University Cancer Center, Augusta, Georgia, USA

## Background

Tumors employ multiple mechanisms to escape immune surveillance and thus hamper cancer immunotherapies. Even when immune responses are generated with tumor vaccines the anti-tumor therapeutic outcome is often not feasible due to tumor-mediated immune suppression. These inhibitory mechanisms involve co-inhibitory receptor-ligand interactions, such as PD-1/PD-L1, secretion of inhibitory molecules, such as TGFβ, IL-10, IDO, and recruitment of suppressive cells, such as regulatory T cells (Treg), myeloid derived suppressor cells (MDSC), etc. Thus, successful cancer immunotherapy requires not only induction and enhancement of effector immune response but also simultaneous targeting of suppressor arm of immune system.

## Materials and methods

Therapeutic and immune efficacy of mono- and combinational immunotherapies were tested in E7 antigen expressing TC-1 mouse tumor model. Tumor growth, survival, as well as peripheral and tumor-infiltrating immune cell profiles after immunotherapy were assessed.

## Results

We developed multiple immune corrective strategies to target various tumor-mediated immune inhibitory mechanisms that enhance anti-tumor immunity and restructure tumor microenvironment to allow effector cells to function potently. We evaluated the immune and therapeutic efficacy of multiple combinational therapies, including blocking and agonist antibodies to co-inhibitory/co-stimulatory molecules, such as PD-1, PD-L1, OX40, CTLA-4, GITR, inhibitors and neutralizing antibodies to inhibitory cytokines/molecules, such as IL-10, TGFβ, IDO, and small molecules for selective inhibition of Tregs. In addition to evaluation of anti-tumor efficacy we also investigated cellular and molecular mechanisms of action for these agents when combined with different vaccine formulations and explored the interactions between compounds within combinational immunotherapies in animal tumor models.

## Conclusion

We are demonstrating the importance of treatment sequence and scheduling when multiple agents are combined which requires full understanding of mechanisms of action for each component and can lead to the successful translation of developed treatment into the clinic.

